# A patient with hereditary angioedema (HAE) with normal C1-INH and SLE with pregnancy

**DOI:** 10.1016/j.jacig.2022.09.005

**Published:** 2022-10-27

**Authors:** Omar S. Taha, Racha Abi Melhem, Yousef Taha, David Meyer, Marc Assaad

**Affiliations:** a Brody School of Medicine, Greenville, NC; b Staten Island University Hospital, Staten Island, NY; c East Carolina University, Greenville, NC

**Keywords:** Hereditary angioedema, SLE, pregnancy, C1-INH, bradykinin receptor blocker, Icatibant, human C1-INH esterase inhibitor

## Abstract

The case of a 24-year-old female patient with hereditary angioedema, a normal C1 esterase inhibitor level, SLE, and pregnancy is reported.

The patient is a 24-year-old White female with a history of recurrent angioedema attacks that started at the age of 12. She was an otherwise healthy child. The swelling affects her face, tongue, lips, throat, and genital area and is accompanied by localized asymmetric nondependent extremity swelling (Fig 1). Abdominal pain, nausea, and vomiting are prominent symptoms during acute episodes. There is no urticaria, pruritus, or erythema marginatum. The attacks usually last for 2 to 4 days and have occurred about 4 times per month, triggered by stress and hormonal fluctuation. Acute episodes have not responded to treatment with oral corticosteroids, antihistamines, tranexamic acid, or fresh frozen plasma. Prophylactic treatment with high-dose antihistamines (4 times the normal dose) for at least 6 months and oral corticosteroids (up to 40 mg per day) were not effective. Tranexamic acid was attempted as prophylactic therapy but was not tolerated owing to abdominal pain and dizziness.

**Fig 1:**
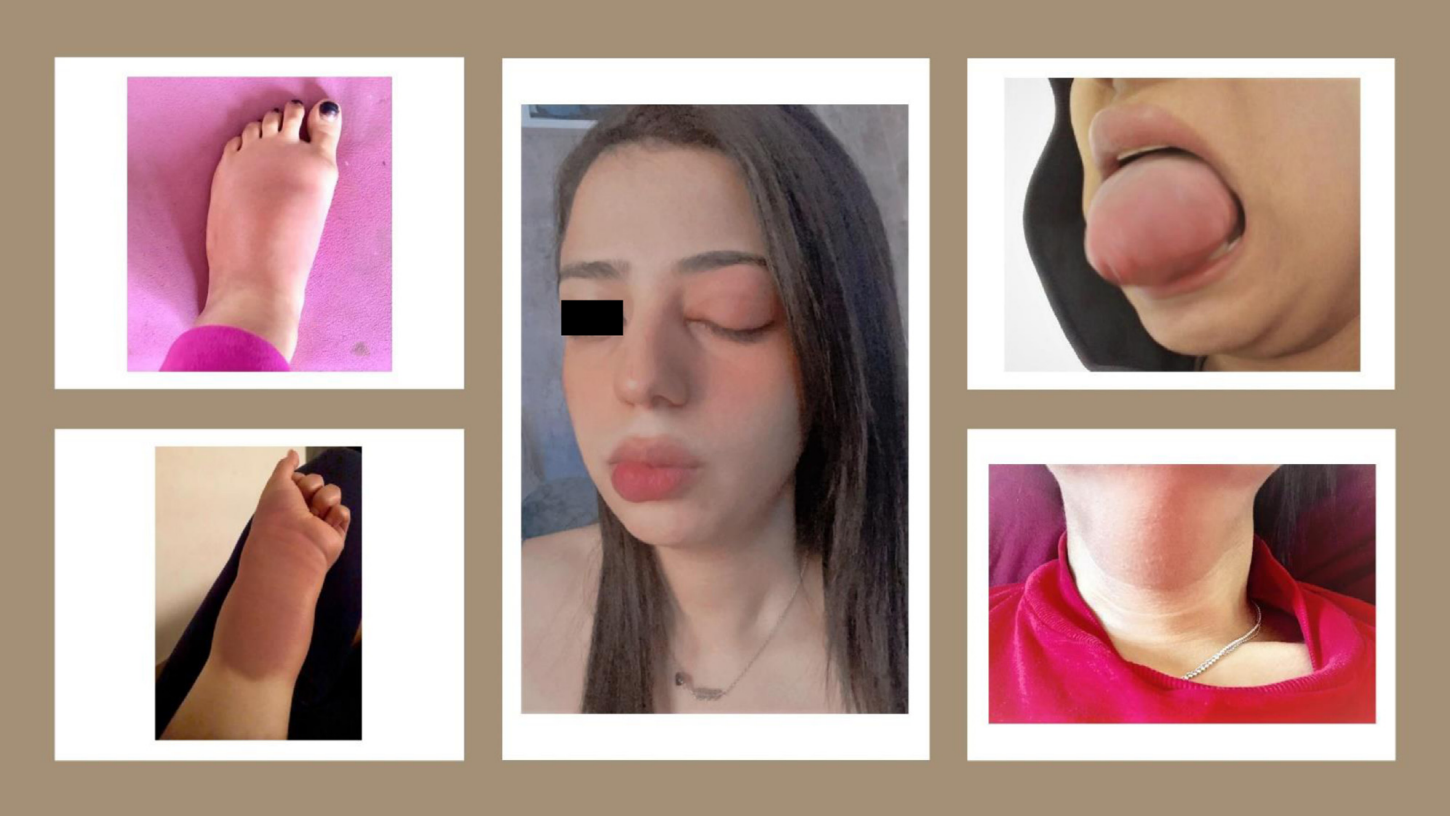
Angioedema at different sites; localized asymmetric swelling of the left foot, left forearm, left eyelid, lips, tongue, and neck.

At the age of 19, the patient started to develop new symptoms of malar rash, photosensitivity with sun exposure, and cold fingertips with color changes. She also had stiff joints of the lower limbs, with knee swelling, microscopic hematuria, and proteinuria. Laboratory workup at that time was positive for autoimmune antibody, anti–double-stranded DNA antibody, and perinuclear antineutrophil cytoplasmic antibody titers (Table I). The diagnosis of SLE was made, and the patient started taking oral corticosteroids. Azathioprine was added later, with improvement of her SLE symptoms. However, she continued to have recurrent episodes of angioedema with the same frequency and severity. Epinephrine, corticosteroids, antihistamines, fresh frozen plasma, and tranexamic acid injections were tried, with little or no improvement.

**Table I:** Laboratory results

Parameter	Result
C1-INH antigen	27 mg/dL (normal range 15-35 mg/dL)∗
C1-INH function	69.7% (normal range 70%-130%) (chromogenic functional assay)∗
C2 complement level	3.1 mg/dL (normal range 1.6-4.0 mg/dL)∗
C4 complement level	32 mg/dL (normal range 10-40 mg/dL)∗
C1q level	18 mg/dL (normal range 12-22 mg/dL), negative C1q binding assay result
ANA	Positive (normal range 1 of 80 homogenous)
Anti-dsDNA	Positive
P-ANCA	Positive
Complete blood count	Normal
Liver and renal profile	Normal
Antiphospholipid antibody	Negative

*ANA*, Antinuclear antibody; *dsDNA*, double-stranded DNA; *P-ANCA*, perinuclear antineutrophil cytoplasmic antibody.

∗ Repeated levels during acute attacks and SLE exacerbation (during the third trimester) were within normal limits.

The patient has no known food or medication allergies and no history of angiotensin-converting enzyme inhibitor use. There is no family history of angioedema. However, her father had a history of rheumatoid arthritis and psoriasis, and her brother is known to have type I diabetes and has recently been diagnosed with Stiff Person syndrome.

## Course during pregnancy

Throughout the patient's pregnancy, her angioedema episodes became more frequent and severe, especially during the third trimester (up to 8 episodes per month). She also developed an SLE flare with proteinuria, hypertension, and low C3 and C4 levels. Of note, the patient did not have access to any of the novel hereditary angioedema (HAE) therapeutic agents until the start of labor.

During latent labor, the patient developed an acute attack of tongue, laryngeal, and vaginal swelling. She was promptly treated with a 30-mg dose of icatibant administered subcutaneously, which aborted the attack within 30 minutes. Of note, there is lack of robust human safety data with regards to icatibant use in pregnancy, and it should be only used if the benefits outweigh the risks to mother and fetus. The patient had a normal vaginal delivery and a healthy full-term male baby. Subsequently, she was given a dose of human C1 esterase inhibitor (C1-INH), 3300 IU subcutaneously, as a start of prophylactic therapy.

She had several acute attacks in the early postpartum period, hence, breast-feeding was stopped, and episodes were treated successfully with icatibant.^1^ She continued taking human C1-INH, 3300 IU twice weekly, and is currently doing significantly better with less frequent and severe episodes (fewer than 1 per month).

## Discussion

The patient's history and clinical presentation suggest a bradykinin-mediated angioedema, and the laboratory workup showed normal C1-INH antigen level and function and normal C2, C4, and C1q levels, suggesting a case of HAE with normal C1-INH levels. It is either a factor XII mutation, other known genetic mutations,^2^ the unknown type,^3^^,^^4^ or nonhistaminergic idiopathic angioedema.^5^ HAE genetic tests are not currently available to the patient.

We report a unique case of possible normal C1-INH HAE and SLE in a pregnant woman. There are case reports of HAE with low complement levels that have evolved into SLE-like disease, although this does not apply to our patient, as she has normal C1-INH and complement levels.^6^ This case possibly provides 2 distinctive, apparently unrelated diseases that did not initiate or affect each other. The use of icatibant was effective in aborting angioedema during labor and the early postpartum period. This case also demonstrates the effectiveness of human C1-INH as a prophylactic therapy, decreasing the frequency and severity of angioedema episodes after labor.
